# Association between frailty and depression among hemodialysis patients: a cross-sectional study

**DOI:** 10.1590/1516-3180.2021.0556.R1.14092021

**Published:** 2022-04-02

**Authors:** Diana Gabriela Mendes dos Santos, Layana Giselly Silva Ferreira, Joice Marques Pallone, Ana Carolina Ottaviani, Ariene Angelini Santos-Orlandi, Sofia Cristina Iost Pavarini, Marisa Silvana Zazzetta, Fabiana de Souza Orlandi

**Affiliations:** I BSc. Master’s Student, Department of Nursing, Universidade Federal de São Carlos (UFSCar), São Carlos (SP), Brazil.; II BSc. Master’s Student, Department of Nursing, Universidade Federal de São Carlos (UFSCar), São Carlos (SP), Brazil.; III Undergraduate Student, Department of Gerontology, Universidade Federal de São Carlos (UFSCar), São Carlos (SP), Brazil.; IV PhD. Postdoctoral Student, Department of Nursing, Universidade Federal de São Carlos (UFSCar), São Carlos (SP), Brazil.; V PhD. Adjunct Professor, Department of Nursing, Universidade Federal de São Carlos (UFSCar), São Carlos (SP), Brazil.; VI PhD. Senior Professor, Department of Gerontology, Universidade Federal de São Carlos (UFSCar), São Carlos (SP), Brazil.; VII PhD. Associate Professor, Department of Gerontology, Universidade Federal de São Carlos (UFSCar), São Carlos (SP), Brazil.; VIII PhD. Associate Professor, Department of Gerontology, Universidade Federal de São Carlos (UFSCar), São Carlos (SP), Brazil.

**Keywords:** Frailty, Renal insufficiency, chronic, Depression, Dialysis, Kidney disease, Frailty syndrome

## Abstract

**BACKGROUND:**

Frailty is consensually understood to be a clinical syndrome in which minimal stressors can lead to negative outcomes such as hospitalization, early institutionalization, falls, functional loss and death. Frailty is more prevalent among patients with chronic kidney disease (CKD), and those on dialysis are the frailest. Depression contributes towards putting patients with CKD into the frailty cycle.

**OBJECTIVE:**

To assess frailty and its relationship with depression among patients with CKD undergoing hemodialysis.

**DESIGN AND SETTING:**

Observational and quantitative cross-sectional study conducted in a renal therapy unit, located in the interior of the state of São Paulo, Brazil.

**METHODS:**

This investigation took place in 2019, among 80 patients. The following instruments were applied: a sociodemographic, economic and health condition characterization and the Subjective Frailty Assessment (SFA) and Patient Health Questionnaire-9 (PHQ-9).

**RESULTS:**

Among the patients, there was higher prevalence of females, individuals with a steady partner and retirees, and their mean age was 59.63 (± 15.14) years. There was high prevalence of physical frailty (73.8%) and depression (93.7%). Depression was associated with frailty, such that patients with depression were 9.8 times more likely to be frail than were patients without depression (odds ratio, OR = 9.80; 95% confidence interval, CI, 1.93-49.79).

**CONCLUSION:**

Based on the proposed objective and the results achieved, it can be concluded that depression was associated with the presence of frailty among patients with CKD on hemodialysis.

## INTRODUCTION

Frailty is consensually understood to be a clinical syndrome in which minimal stressors can lead to negative outcomes such as hospitalization, early institutionalization, falls, functional loss and death.^1^ There are several models for frailty, but the two that have been most widely used are the deficit model, developed by Mitnitski, Mogilner and Rockwood,^2^ and the phenotype-based model, developed by Fried et al.^3^

Because of the high incidence and prevalence of physical and cognitive impairment among patients with chronic kidney disease (CKD), these individuals are more likely to develop frailty early.^4^ According to a systematic review carried out by Chowndhuty et al.,^5^ frailty is very prevalent in patients with CKD, and those on dialysis are the frailest. Furthermore, frailty has been shown to be associated with increased risk of hospitalization and mortality among patients with CKD. An association between frailty and higher risk of mortality among patients seen at rapid diagnostic clinics (RDCs) has been found in other studies in the literature.^4,6,7^

According to Wu et al.,^6^ in addition to sociodemographic factors and health issues, depression contributes towards putting patients with CKD into the frailty cycle. A study by Perez et al.^7^ among frail and non-frail adults with diabetes mellitus (type 1 and type 2) and with chronic kidney disease (stages 1 to 5) demonstrated that frail participants presented depression more often (P ≤ 0.005) than did the non-frail participants. In addition, depression has been found to be the most recurrent psychiatric condition among patients with CKD, with a rate of 20 to 30% among patients on hemodialysis.^8^

## OBJECTIVE

The aim of the present study was to assess frailty and its association with depression among patients with CKD undergoing hemodialysis.

## METHODS

### Design

The present study was of observational and quantitative cross-sectional nature. The investigation took place in 2019, at a dialysis service located in the interior of the state of São Paulo, Brazil.

### Sample

The final sample was 80 patients who met the following inclusion criteria: having a medical diagnosis of CKD, being on hemodialysis and possessing preserved oral communication. The exclusion criterion was presentation of dementia that could be verified in the medical records. An initial non-probabilistic convenience sample of 180 patients was used; seventy patients were excluded from the study, 46 did not meet the study’s eligibility criteria and 64 did not agree to participate in the study.

### Data collection

The data collection process took place as follows. At the initial contact with the patients, the research project was explained to them and they were invited to participate in the study. Patients who agreed to participate signed a free and informed consent statement. At their next hemodialysis session, and specifically within the first two hours (during which patients present with fewer hemodynamic changes), evaluations were started using a sociodemographic, economic and health condition characterization and the Subjective Frailty Assessment (SFA) and Patient Health Questionnaire-9 (PHQ-9).

The sociodemographic, economic and health condition characterization consisted of asking about the following characteristics: sex, age, marital status, ethnicity, comorbidities, education level, income, time on hemodialysis and number of medications.

The SFA was validated in Brazil by Nunes,^9^ based on the components of the frailty phenotype proposed by Fried et al.,^10^ which was considered to be the gold standard. The frailty phenotype consists of the following five criteria: unintentional weight loss, reported fatigue, reduced grip strength, reduced walking speed and low physical activity. Nunes^9^ elaborated and validated items for subjective evaluation, especially through dichotomous questions about each component. The SFA can have a total score of 5 points, and individuals are considered non-frail if they do not have any of the criteria evaluated. They are considered pre-frail if they have one or two of these components and frail if they have three or more components.

The PHQ-9 is a screening scale for depression that was validated by Kroenke, Spitzer and Williams^11^ and adapted for use in Brazil by Santos et al.^12^ It is an instrument with nine questions based on the clinical diagnostic criteria for depressive episodes (Diagnostic and Statistical Manual of Mental Disorders, fourth edition, DSM-IV), which form the evaluation module for depressive disorders in the Patient Health Questionnaire (PHQ). The symptoms evaluated are the following: depressed mood; anhedonia (loss of interest or pleasure in doing things); problems with sleep, tiredness or lack of energy; changes in appetite or weight; feelings of guilt or worthlessness; problems with concentration; feelings of slowness or restlessness; and thoughts of suicide. The frequency of each symptom over the past two weeks is assessed on a Likert scale from 0 to 3 corresponding to the answers “no days”, “less than a week”, “more than a week” and “almost every day”, respectively. The questionnaire also includes a tenth question that assesses the interference of these symptoms in performing daily activities, such as working and studying. If the final score reaches 5, the individual is characterized as having mild depression; 10, moderate; 15, moderately severe; and 20 or more, severe.

### Data analysis

The statistical treatment of the data was performed with support from the Statistical Package for the Social Sciences (SPSS) software, version 22.0 (IBM Corporation, Armonk, New York, United States). Descriptive analyses were performed, with preparation, including central trend data (average, minimum and maximum) and dispersion measurements (standard deviation). The Kolmogorov-Smirnov test was performed to ascertain whether the data showed normal distribution. This showed that nonparametric tests needed to be used.

To compare continuous variables according to the level of physical frailty, with subjective frailty assessment (non-frail, pre-frail or frail), the Kruskal-Wallis test was used. To compare categorical variables according to the level of physical frailty, with subjective frailty assessment (non-frail, pre-frail or frail), the Pearson test was adopted. The significance level for the statistical tests was taken to be 5% (P ≤ 0.05).

To investigate the association between depression and frailty, multivariate logistic regression analysis was performed. Initially, univariate logistic regression analyses were performed using sociodemographic, economic and health condition variables. Statistically significant variables (P < 0.25) in the univariate regression analyses were selected for the multivariate analysis. The model was adapted for sex, age and education. The respective odds ratios (OR) and 95% confidence intervals (CI) were calculated.

### Ethical considerations

The protocol was approved by the ethics committee of our institution (CAAE: 18828419.0.0000.5504; number 3.535.236; date: August 27, 2019). Participants needed to sign an informed consent statement before entering the study.

## RESULTS

Regarding frailty assessed according to the SFA scale, it was found that only four participants were non-frail, while 17 (21.2%) were pre-frail and 59 (73.8%) were frail. The sociodemographic, economic and comorbidity profile did not show any statistically relevant difference between the levels of frailty. The majority of the participants were female, white and retired, and had a steady partner. Their average age was less than 61 years; their average schooling level did not exceed 8.5 years; and their average per capita income did not exceed 1.5 minimum monthly wages (Table 1).

**Table 1.** Sociodemographic variables, economic characteristics, comorbidities and depression measured through the patient health questionnaire-9 (PHQ-9), according to levels of frailty. São Carlos (SP), Brazil, 2019 (n = 80)

Variable	Categories	Non-frail (n = 4)	Pre-frail (n = 17)	Frail (n = 59)	P-value
**Sex**	Male	3	8	25	0.44
	Female	1	9	34	
**Ethnicity**	White	4	13	35	0.52
	Black	0	2	18	
	Brown	0	2	5	
	East Asian	0	0	1	
**Marital status**	With a fixed partner	4	10	38	
	No fixed partner	0	7	20	0.59
**Occupation**	Retired	4	8	45	
	Absent*	0	4	6	0.11
	Housewife	0	3	7	
	Other	0	2	0	
**Comorbidities**	Diabetes				0.67
	Yes	2	12	36	
	No	2	5	23	
	Hypertension				
	Yes	2	7	26	0.95
	No	2	10	33	
	Other types of comorbidities				
	Yes	3	17	52	0.21
	No	1	0	7	
**Variable**	**Categories**		**Mean**		**P-value**
Age**	Non-frail (n = 4)		60.25		
	Pre-frail (n = 17)		55.88		0.59
	Frail (n = 59)		60.66		
Education level**	Non-frail (n = 4)		8.50		0.15
	Pre-frail (n = 17)		7.24		
	Frail (n = 59)		6.37		
Income***	Non-frail (n = 4)		1,226.75		
	Pre-frail (n = 17)		996.88		0.20
	Frail (n = 59)		930.50		
**Number of medications**	Non-frail (n = 4)		6,50		
	Pre-frail (n = 17)		6.06		0.57
	Frail (n = 59)		6.41		
**Depression (PHQ-9)**	Non-frail (n = 4)		5.00		
	Pre-frail (n = 17)		8.88		0.001
	Frail (n = 59)		15.92		

*Absent from work, as approved by the National Institute of Social Security. **In years; ***In reais; Kruskal-Wallis test; Pearson’s test.

Regarding the comorbidities of diabetes and hypertension, Table 1 shows that most participants reported having these comorbidities, regardless of their level of frailty. The number of continuous-use medications was 6.50 for non-frail, 6.06 for pre-frail and 6.41 for frail individuals.

Regarding depression, it was noted that overall, 83.7% of the participants had depression, at levels ranging from mild to severe. When compared according to frailty levels, there was a statistically significant difference between the groups. Non-frail and pre-frail patients had mean scores of 5 and 8.88, respectively, thus characterizing a mild degree of depression. Frail participants, on the other hand, had an average score of 15.92, thus characterizing moderately severe depression, according to the PHQ-9 instrument (Table 1).

In the analysis on depression as a predictor of frailty, it was noted that depression was associated with frailty. Patients with depression were 9.8 times more likely to be frail than were patients without depression (OR = 9.80; 95% CI, 1.93-49.79). For the other variables analyzed (sex, age and education level), there was no statistically relevant relationship (Table 2).

**Table 2.** Multivariate logistic regression analysis on frailty (n = 80)

Frailty			
Variables	P-value	OR*	95% CI
Sex**	0.105	2.27	1.93-49.79
**Age**	0.601	1.01	0.97-1.05
**Education level**	0.574	0.96	0.83-1.11
Depression***	0.006	9.80	1.93-49.79

*Risk ratio for frailty/subjective frailty assessment; **women; ***presence of depression. OR = odds ratio; CI = confidence interval.

## DISCUSSION

In the present study, among the sociodemographic, economic and health condition characteristics of the participants, female sex was more prevalent, the mean age was 59.63 years and the mean schooling level was 6.66 years. It was noted that 83.7% of the patients presented depression. This profile has also been found in other studies conducted in Brazil and in other countries among patients with CKD.^4,13,14^

Assessing the occurrence rates of frailty and depression syndromes is challenging given the diversity of models and measurements that have been used in studies to identify their prevalences. In this, the heterogeneity of the conditions themselves needs to be borne in mind: their expression of signs and symptoms varies according to age and socioeconomic context.^5^

With regard to frailty, 21.3% of the patients were considered pre-frail in the present study. However, in the study by Gesualdo et al., in which the objective was to evaluate frailty and identify its associated factors among adults and elderly people with CKD who were undergoing hemodialytic treatment, most of the adults were pre-frail (54.84%). Their study corroborates the findings of the present study regarding the prevalence of frailty.^4^

Another study that addressed factors associated with frailty was conducted by Duarte et al.^17^ In that study, the aim was to describe the prevalence of frailty among elderly people, in order to analyze associated factors and the evolution of the syndrome. A total of 1,399 older adults aged 60 years or older, living in the state of São Paulo, Brazil, participated in the study. Frailty syndrome was evaluated based on the phenotype proposed by Fried et al.,^10^ composed of five components: unintentional weight loss, self-reported fatigue, reduction in strength, low walking speed and low level of physical activity. Duarte et al.^17^ found that out of the total number of elderly subjects (n = 1,399), 8.5% were frail and presented factors associated with age, functional impairment, cognitive decline, hospitalization and multimorbidity. Over a four-year period, 3.3% of the non-frail and 14.7% of the pre-frail elderly people became frail. In the present study, we found that the prevalence of frailty among the patients was 73.8%.

Although most definitions of frailty focus primarily on physical and biological indicators of vulnerability, it is significant to consider the risk of adverse health outcomes associated with mental disorders. As well as frailty, depression among the elderly has been characterized in terms of diminished reserve capacity, thus representing a lack of resources to respond to stressors.^15^ Hence, depression can serve to accentuate physical frailty: not because it is associated with any disease process or model of frailty in particular, but because it represents a lack of psychological and social coping resources.^15,16^

The association between frailty and depression demonstrated in the present study was also found in other studies, such as the one by Tavares et al.^17^ In that study, the aim was to describe the socioeconomic variables of older adults with indications of depression according to sex; investigate the association between frailty status and sex; and describe the component of the frailty phenotype most impacted among elderly people with indications of pre-frail and frail depression. A total of 418 elderly people with indications of depression living in the city of Uberaba, Minas Gerais, Brazil, participated in that study. The results showed that the prevalence of frailty among older adults with indications of depression was 27.8%, while 51.7% presented pre-frailty. That study corroborated the findings of the present study, in which depression was associated with frailty, thus indicating that patients with depression were 9.8 times more likely to be frail than were patients without depression. In the light of this exposure, it is possible to infer that patients with CKD who are undergoing hemodialysis treatment are more vulnerable to becoming affected by depressive symptoms.

Another study that addressed depression in CKD patients was conducted by Pretto.^18^ The aim in that study was to investigate associations shown by sociodemographic variables, clinical factors, lifestyle habits and functional capacity in relation to indications of depression among chronic renal patients on hemodialysis. The study participants were 238 patients, who were attending the reference renal units for the Northwest and Missions regions of Rio Grande do Sul, Brazil. Beck’s Depression Inventory (BDI) was used to assess depression. It was found that the prevalence of symptoms indicative of depression among the patients was 60.3% (111): mild in 36.4% (67), moderate in 22.3% (41) and severe in 1.6% (3).

Depression scores are three to four times higher among patients with CKD than in the general population and two to three times higher than among people with other chronic diseases. In the present study, it was found that 83.7% of the patients had depressive symptoms, i.e. more than half of the patients presented depression, just as in the study by Pretto.^18^

Nursing plays an important role in this scenario. Nurses are the professionals who establish greatest contact and bonding with these patients, given the long periods of time spent with them. 

Health education plays a fundamental role in promoting quality of life for patients affected by CKD. Moreover, this raises awareness among patients, family members and the nursing team, thereby stimulating changes to behavioral strategies.

Future research should continue to explore the reasons for comorbidities and the implications of comorbidities in predicting adverse health outcomes. Therapies to combat depression can prevent or mitigate the symptoms of frailty.

The limitation of the present study was that its sample was selected for convenience.

## CONCLUSION

From the proposed objective of this study and the results achieved, it can be concluded that depression was associated with the presence of frailty among patients with CKD who were undergoing hemodialysis. Thus, it is important to highlight the need for early screening of frailty in this population.

In addition, there is an urgent requirement to create healthcare policies that meet the social and psychological needs of these patients, given that this is a predictable and preventable syndrome. In addition, professionals must pay attention to the signs and symptoms of depression that can lead patients to frailty, in addition to other outcomes.
